# Different implications of daytime and nighttime heart rate variability on total burden of cerebral small vascular disease in patients with nondisabling ischemic cerebrovascular events

**DOI:** 10.3389/fcvm.2024.1434041

**Published:** 2024-10-21

**Authors:** Zhixiang Zhang, Yijun Lv, Qian Wang, Yan Wang, Min Zhang, Yongjun Cao

**Affiliations:** ^1^ Department of Neurology, The Second Affiliated Hospital of Soochow University, Suzhou, China; ^2^ Department of Neurology, The Affiliated Changzhou Second People’s Hospital of Nanjing Medical University, Changzhou Medical Center of Nanjing Medical University, Changzhou, China

**Keywords:** nondisabling ischemic cerebrovascular events, cerebral small vessel disease, total burden, heart rate variability, daytime, nighttime

## Abstract

**Objective:**

This study aimed to explore the relationship between total burden of cerebral small vessel disease (CSVD) and daytime and nighttime heart rate variability (HRV) parameters.

**Method:**

Consecutive patients with nondisabling ischemic cerebrovascular events were recruited from the cerebrovascular disease clinic of Changzhou Second People's Hospital between January 2022 and June 2023. A total of 144 enrolled participants were divided into a mild CSVD group (74 patients) and a moderate-to-severe CSVD group (70 patients) based on total burden of CSVD. Various HRV parameters measured during 24-h, 4-h daytime, and 4-h nighttime periods (including natural log–transformed [ln] root mean square of successive RR interval differences [RMSSD], ln absolute power of the high-frequency band [0.15–0.4 Hz] [HF], ln absolute power of the low-frequency band [0.04–0.15 Hz][LF], and LF-to-HF ratio [LF/HF]) were then assessed in the 2 groups. Spearman correlation analysis was used to assess the correlation between total burden of CSVD and HRV parameters. HRV parameters with *P*-value < 0.05 in correlation analysis were included in the multivariable logistic regression analysis, and restricted cubic spline analysis was performed to assess dose-response relationships.

**Results:**

Daytime 4-h lnRMSSD (r = –0.221; *P* = 0.008) and 4-h lnHF (r = –0.232; *P* = 0.005) were negatively correlated with total burden of CSVD, and daytime 4-h lnLF/HF (r = 0.187; *P* = 0.025) was positively correlated with total burden of CSVD. There was no correlation between nighttime HRV parameters and total burden of CSVD. After adjustments were made for potential confounders, daytime 4-h lnRMSSD (OR = 0.34; 95% CI: 0.16–0.76), 4-h lnHF (OR = 0.57; 95% CI: 0.39–0.84), and 4-h lnLF/HF (OR = 2.12; 95% CI: 1.18–3.82) were independent predictors of total burden of CSVD (all *P* < 0.05). S-shaped linear associations with moderate-to-severe total burden of CSVD were seen for daytime 4h-lnRMSSD (*P* for nonlinearity = 0.543), 4-h lnHF (*P* for nonlinearity = 0.31), and 4-h lnLF/HF (*P* for nonlinearity = 0.502).

**Conclusion:**

Daytime parasympathetic HRV parameters are independent influencing factors of total burden of CSVD and may serve as potential therapeutic observation indicators for CSVD.

## 1 Introduction

Nondisabling ischemic cerebrovascular events, including transient ischemic attacks and mild acute ischemic strokes, are closely related to cerebral small vessel disease (CSVD) (1, 2). CSVD refers to a range of pathologic processes that damage small end arteries, arterioles, venules, and capillaries (3). CSVD is a global rather than a focal disease, and its severity can be comprehensively evaluated by assessing the total disease burden **(**4, 5). In general, CSVD is defined by the presence of specific features on magnetic resonance (MR) images of the brain, including white matter hyperintensities (WMHs), lacunes, enlarged perivascular spaces, cerebral microbleeds, and brain atrophy (6). Previous studies have identified several imaging-based biomarkers as potential therapeutic targets for CSVD (7–10).

Heart rate variability (HRV), which refers to the fluctuation in time intervals between heartbeats, is controlled by heart-brain interactions and has been shown to index the function of the autonomic nervous system (11). HRV is divided into three categories based on recording duration: long-term [≥24 h (hereafter referred to as “24-h HRV”)], short-term (∼5 min), and ultra–short-term (<5 min) (12). Previous research has shown that 24-h HRV is related to prognosis in patients with cerebrovascular disease and cognitive impairment (13, 14). Additionally, several recent studies have suggested that 24-h HRV is associated with total disease burden in patients with CSVD (15–17). Therefore, 24-h HRV is considered a potential therapeutic target for CSVD.

Previous studies have shown that HRV can vary between day and night and between wake and sleep states (18, 19). Aggregated values of 24-h HRV may fail to account for these differences. The goal of this study, therefore, was to explore the differences in correlations between the severity of CSVD and HRV during day (wake) and night (sleep), with the aim of elucidating the relationship between the autonomic nervous system and CSVD and thus providing further evidence regarding potential therapeutic observation indicators.

## 2 Methods

### 2.1 Study participants

The participants in this study were consecutively recruited from the cerebrovascular disease clinic of the Affiliated Changzhou Second People's Hospital of Nanjing Medical University between January 2022 and June 2023. Patients were included if they were aged ≥40 years; if they had experienced a nondisabling ischemic cerebrovascular event [including a transient ischemic attack or an acute ischemic stroke (National Institutes of Health Stroke Scale score ≤4)] 3 months ago; if their current Modified Rankin Scale score was 0 or 1; and if they demonstrated ≥1 MR imaging feature of CSVD. Patients were excluded if they had an incomplete dynamic electrocardiogram recording or non-sinus rhythm such as atrial fibrillation or other arrhythmia; if their sleep diary indicated that they were in a nonsleeping state between 11 PM and 3 AM; if they regularly used beta-blockers; if they drank alcohol, coffee, or tea or smoked cigarettes during the dynamic electrocardiogram examination; if they had experienced acute cerebrovascular disease within the previous 3 months; if they had cardiovascular conditions such as severe ischemic heart disease, severe valvular heart disease, or heart failure; and if they had been diagnosed with sleep apnea syndrome, depression, or any other disease known to affect the autonomic nervous system.

This study was approved by the Ethics Committee of the Affiliated Changzhou Second People's Hospital of Nanjing Medical University (approval number: 2021KY312-01) and was conducted according to the Declaration of Helsinki. All study participants provided written informed consent.

### 2.2 Clinical data collection

At baseline, bloodwork was performed to assess patients’ serum creatinine, uric acid, lipid, homocysteine, glucose, and HbA1c levels, and blood pressure was measured in all patients. Data were also collected regarding patients’ age; sex; tobacco and alcohol use (with current use defined as use within the past month); and history of hypertension (defined as baseline systolic blood pressure ≥140 mmHg, diastolic blood pressure ≥90 mmHg, or previous diagnosis of hypertension), diabetes mellitus (defined as fasting blood glucose ≥7.0 mmol/L and HbA1c ≥ 6.5%, or previous diagnosis of diabetes mellitus), and hyperlipemia (defined as total cholesterol ≥5.2 mmol/L, low-density lipoprotein-C ≥ 3.36 mmol/L, or previous diagnosis of hyperlipidemia).

### 2.3 Measurement of HRV

All patients underwent 24-h Holter monitoring (C7201A092, GE) and were required to begin sleep at 10 PM during the study period. To determine whether there was a diurnal difference in the correlation between HRV and CSVD, we selected representative time periods of the same duration during day and night. The period of 11 PM to 3 AM was selected as the nighttime measurement period to ensure that most of the participants would be in a sleep state. Correspondingly, the period of 11 AM to 3 PM was selected as the daytime measurement period; during this period, participants were required to remain awake and engage in daily activities (but avoid strenuous exercise).

We assessed 24-h, daytime 4-h, and nighttime 4-h HRV using time-domain and frequency-domain measurements (12), which included standard deviation of NN intervals (SDNN), root mean square of successive RR interval differences (RMSSD), absolute power of the low-frequency band (LF), absolute power of the high-frequency band (HF), and ratio of LF-to-HF power (LF/HF). All participants’ data were manually reviewed by the same electrocardiogram specialist.

### 2.4 Measurement of the severity of CSVD

All patients underwent structural imaging on a 3.0 T MR scanner, including T1- and T2-weighted sequences, a fluid-attenuated inversion recovery sequence, diffusion-weighted imaging with apparent diffusion coefficient, and a susceptibility-weighted imaging sequence. All MR data were evaluated by two well-trained [8 years of experience] attending neurologists who were blinded to the clinical data. If their conclusions were inconsistent, a senior neurologist [20 years of experience] assisted in the evaluation.

The neuroimaging markers of CSVD, including WMHs, enlarged perivascular spaces, lacunes, and cerebral microbleeds, were analyzed based on the Standards for Reporting Vascular Changes on Neuroimaging guidelines (20), and the total burden of CSVD was scored on a scale ranging from 0 to 4 by assessing the presence of each of these neuroimaging markers (21). The presence of WMH (1 point) was defined as the presence of either confluent deep WMHs (Fazekas score 2 or 3) or irregular periventricular WMHs extending into the deep white matter (Fazekas score 3) (22). The presence of enlarged perivascular spaces (1 point) was defined as the presence of moderate to severe enlarged perivascular spaces (semiquantitative grade 2−4) in the basal ganglia (23). The presence of lacunes (1 point) was defined as the presence of ≥1 lacunes, and the presence of cerebral microbleeds (1 point) was defined as the presence of any cerebral microbleed.

### 2.5 Statistical analysis

All patients were divided into groups based on the total burden of CSVD: mild (0–1 points) or moderate to severe (2–4 points) (24). Data are presented as mean ± standard deviation (SD) or median with interquartile range (IQR) for continuous variables and as absolute with percentage for categorical variables. Natural log transformation was performed for the HRV parameters (lnHRV) to meet a normal distribution (25, 26).

In analyses of baseline characteristics and HRV parameters, statistical differences between the mild and moderate-to-severe groups were assessed using a *t*-test or Mann-Whitney *U*-test for continuous variables and a chi-square or Fisher test for categorical variables. Associations between HRV parameters and total burden of CSVD were assessed using Spearman correlation analysis. HRV parameters demonstrating *P*-values < 0.05 in the correlation analysis were included in the multivariable logistic regression analysis. To eliminate underlying confounding bias, three models were constructed: Model 1 was adjusted for age and sex; Model 2 was adjusted for confounders at *P* < 0.05 in the correlation analysis; and Model 3 was adjusted for all baseline characteristics with the exception of lipid indicators.

After adjustments were made for potential confounders in model 3, a restricted cubic spline analysis was performed to explore the dose-response relationships between HRV and total burden of CSVD. Reference values [odds radio (OR) = 1] were set at the 50th percentile, and knots were set at the 5th, 35th, 65th, and 95th percentiles of ln-HRV (27, 28).

The significance level was set at *P* <0.05 (two-sided). All analyses were performed using R Statistical Software (version 4.2.2, http://www.R-project.org, The R Foundation) and the Free Statistics analysis platform (version 1.8).

## 3 Results

### 3.1 Baseline characteristics of study participants

A total of 144 participants were enrolled in the study, including 74 patients (51.4%) in the mild CSVD group and 70 patients (48.6%) in the moderate-to-severe CSVD group. The baseline characteristics of the study participants are shown in Table 1. Participants in the moderate-to-severe group were generally older than those in the mild group (median, 73 y vs. 69 y; *P* = 0.003). The moderate-to-severe group had a higher proportion of male patients (52% vs. 33%; *P* < 0.001) and a higher proportion of patients with hypertension (64% vs. 56%; *P* = 0.011). Patients in the moderate-to-severe group also demonstrated higher levels of serum creatinine (mean, 81.3 µmol/L vs. 71.9 µmol/L; *P* = 0.039), serum uric acid (mean, 347.1 µmol/L vs. 309.6 µmol/l; *P* = 0.019), and homocysteine (mean, 18.6 µmol/L vs. 14.1 µmol/L; *P* = 0.011). There were no significant differences between the groups in the other baseline characteristics.

**Table 1:** Baseline characteristics of study participants.

Variable	Total (*n* = 144)	Total burden of CSVD		*P*-value
		0–1 point (*n* = 74)	2–4 points (*n* = 70)	
Age, years	71 (63, 76)	69 (61, 74)	73 (66, 79)	0.003
Male	85 (59.0)	33 (44.6)	52 (74.3)	<0.001
Medical history				
Hypertension	120 (83.3)	56 (75.7)	64 (91.4)	0.011
Diabetes mellitus	47 (32.6)	27 (36.5)	20 (28.6)	0.311
Hyperlipemia	39 (27.1)	22 (29.7)	17 (24.3)	0.462
Current smoker	41 (28.5)	18 (24.3)	23 (32.9)	0.257
Current alcohol user	25 (17.4)	12 (16.2)	13 (18.6)	0.709
Laboratory tests				
Total cholesterol, mmol/L	4.2 ± 1.0	4.2 ± 0.9	4.1 ± 1.0	0.599
Triglycerides, mmol/L	1.6 ± 0.8	1.5 ± 0.9	1.6 ± 0.8	0.967
HDL-C, mmol/L	1.1 ± 0.3	1.1 ± 0.3	1.1 ± 0.3	0.105
LDL-C, mmol/L	2.4 ± 0.8	2.5 ± 0.7	2.4 ± 0.9	0.716
Creatinine, µmol/L	77.4 ± 32.7	71.9 ± 27.0	83.1 ± 37.2	0.039
Uric acid, µmol/L	327.8 ± 96.4	309.6 ± 91.9	347.1 ± 98.1	0.019
Homocysteine, µmol/L	16.3 ± 10.6	14.1 ± 7.2	18.6 ± 13.0	0.011
Glycosylated hemoglobin,%	6.6 ± 1.7	6.6 ± 1.7	6.5 ± 1.7	0.91

Data are median (Q1, Q3), *n* (%), or mean ± SD.

CSVD, cerebral small vessel disease; HDL-C, high-density lipoprotein cholesterol; LDL-C, low-density lipoprotein cholesterol.

### 3.2 HRV parameters in study participants

The HRV parameters for the two groups are shown in Table 2. No significant differences were observed between the groups with respect to 24-h HRV parameters, nighttime 4-h HRV parameters, daytime 4-h lnSDNN, or 4-h lnLF. Participants in the moderate-to-severe group had lower daytime 4-h lnRMSSD (mean, 3.6 ms vs. 3.8 ms; *P* = 0.02) and 4-h lnHF (mean, 5.4 ms^2^ vs. 5.9 ms^2^; *P* = 0.013) and higher daytime 4-h lnLF/HF (mean, 0.2 vs. −0.1; *P* = 0.019) compared with patients in the mild group.

**Table 2:** HRV parameters of participants.

Variable	Total (*n* = 144)	Total burden of CSVD		*P*-value
		0–1 point (*n* = 74)	2–4 points (*n* = 70)	
24-h HRV				
lnSDNN, ms	4.6 ± 0.3	4.7 ± 0.3	4.6 ± 0.3	0.288
lnRMSSD, ms	3.6 ± 0.4	3.7 ± 0.4	3.6 ± 0.5	0.264
lnLF, ms^2^	5.7 ± 0.9	5.8 ± 0.8	5.7 ± 1.0	0.689
lnHF, ms^2^	5.7 ± 1.0	5.8 ± 0.8	5.6 ± 1.1	0.228
lnLF/HF	0.0 ± 0.6	0.0 ± 0.5	0.1 ± 0.7	0.191
Daytime 4-h HRV				
lnSDNN, ms	4.4 ± 0.3	4.4 ± 0.3	4.3 ± 0.3	0.243
lnRMSSD, ms	3.7 ± 0.5	3.8 ± 0.5	3.6 ± 0.5	0.02
lnLF, ms^2^	5.7 ± 1.0	5.8 ± 1.0	5.6 ± 1.1	0.347
lnHF, ms^2^	5.6 ± 1.1	5.9 ± 1.0	5.4 ± 1.1	0.013
lnLF/HF	0.1 ± 0.7	−0.1 ± 0.7	0.2 ± 0.7	0.019
Nighttime 4-h HRV				
lnSDNN, ms	4.3 ± 0.4	4.3 ± 0.3	4.3 ± 0.4	0.889
lnRMSSD, ms	3.5 ± 0.5	3.5 ± 0.4	3.5 ± 0.6	0.494
lnLF, ms^2^	5.7 ± 0.9	5.7 ± 0.9	5.7 ± 1.0	0.692
lnHF, ms^2^	5.6 ± 1.0	5.7 ± 0.9	5.5 ± 1.2	0.334
lnLF/HF	0.1 ± 0.7	0.0 ± 0.6	0.1 ± 0.8	0.375

Data are mean ± SD.

CSVD, cerebral small vessel disease; HRV, heart rate variability; RMSSD, root mean square of successive RR interval differences; SDNN, standard deviation of NN intervals; HF, absolute power of the high-frequency band (0.15–0.4 Hz); LF, absolute power of the low-frequency band (0.04–0.15 Hz); LF/HF, ratio of LF-to-HF power.

### 3.3 Relationships between HRV parameters and total burden of CSVD

Spearman correlation analysis results are shown in Table 3. The daytime 4-h lnRMSSD (r = –0.221; *P* = 0.008) and 4-h lnHF (r = –0.232; *P* = 0.005) were negatively correlated with total burden of CSVD, and 4-h lnLF/HF (r = 0.187; *P* = 0.025) was positively correlated with total burden of CSVD.

**Table 3:** Correlation between HRV parameters and total burden of CSVD.

Variable	r_s_ value	*P*-value
24-h HRV		
lnSDNN	−0.078	0.355
lnRMSSD	−0.134	0.110
lnLF	−0.064	0.450
lnHF	−0.153	0.066
lnLF/HF	0.108	0.198
Daytime 4-h HRV		
lnSDNN	−0.108	0.197
lnRMSSD	−0.221	0.008
lnLF	−0.092	0.273
lnHF	−0.232	0.005
lnLF/HF	0.187	0.025
Nighttime 4-h HRV		
lnSDNN	−0.021	0.805
lnRMSSD	−0.104	0.214
lnLF	−0.049	0.559
lnHF	−0.129	0.124
lnLF/HF	0.085	0.313

CSVD, cerebral small vessel disease; HRV, heart rate variability; RMSSD, root mean square of successive RR interval differences; SDNN, standard deviation of NN intervals; HF, absolute power of the high-frequency band (0.15–0.4 Hz); LF, absolute power of the low-frequency band (0.04–0.15 Hz); LF/HF, ratio of LF-to-HF power.

### 3.4 Associations between daytime 4-h HRV and total burden of CSVD

Daytime 4-h HRV parameters with *P*-values < 0.05 were sequentially included in the multivariable logistic regression model. After adjustments were made for all potential confounders (including age, sex, hypertension, diabetes mellitus, hyperlipemia, current smoker, current alcohol user, serum uric acid level, serum creatinine level, and homocysteine level), 4-h lnRMSSD [OR = 0.34; 95% confidence interval (CI): 0.16–0.76], 4-h lnHF (OR = 0.57; 95% CI: 0.39–0.84), and 4-h lnLF/HF (OR = 2.12; 95% CI: 1.18–3.82) were independently associated with total burden of CSVD (all *P*-values < 0.05) (Table 4).

**Table 4:** Logistic regression analysis of daytime 4-h HRV parameters.

Daytime 4-h HRV parameter	Model 1		Model 2		Model 3	
	OR (95% CI)	*P*-value	OR (95% CI)	*P*-value	OR (95% CI)	*P*-value
lnRMSSD	0.35 (0.17–0.74)	0.006	0.37 (0.17–0.79)	0.010	0.34 (0.16–0.76)	0.008
lnHF	0.59 (0.41–0.85)	0.005	0.60 (0.42–0.87)	0.006	0.57 (0.39–0.84)	0.004
lnLF/HF	2.17 (1.22–3.88)	0.009	2.15 (1.2–3.86)	0.010	2.12 (1.18–3.82)	0.012

Model 1 was adjusted for age and sex. Model 2 was adjusted for age, sex, hypertension, serum uric acid level, serum creatinine level, and homocysteine level. Model 3 was adjusted for age, sex, hypertension, diabetes mellitus, hyperlipemia, current smoker, current alcohol user, serum uric acid level, serum creatinine level, and homocysteine level.

HRV, heart rate variability; CI, confidence interval; RMSSD, root mean square of successive RR interval differences; HF, absolute power of the high-frequency band (0.15–0.4 Hz); LF, absolute power of the low-frequency band (0.04–0.15 Hz); LF/HF, ratio of LF-to-HF power.

### 3.5 Dose-response relationships between daytime 4-h HRV and total burden of CSVD

**Figure 1:**
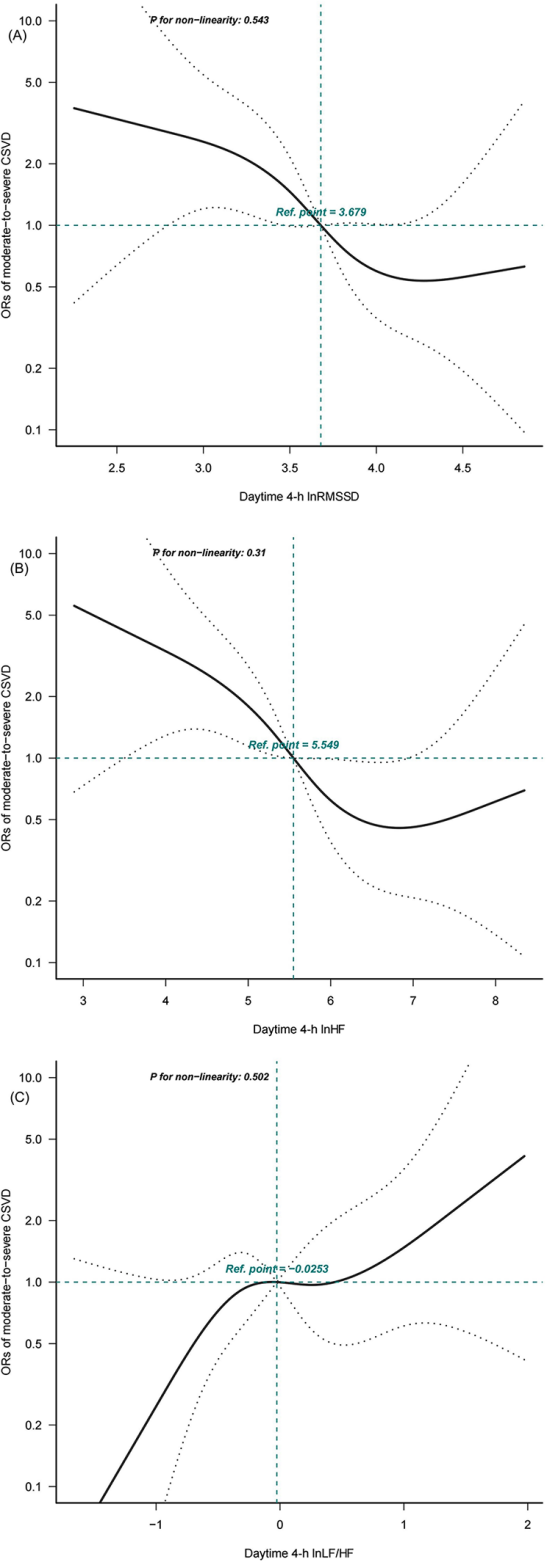
Dose-response relationships between daytime 4-h heart rate variability (HRV) parameters and cerebral small vessel disease (CSVD) calculated using restricted cubic spline analysis. Adjusted binary logistic regression models of restricted cubic splines show odds radios (ORs) for moderate-to-severe CSVD based on **(A)** daytime 4-h ln root mean square of successive RR interval differences (RMSSD), **(B)** daytime 4-h ln absolute power of the high-frequency band (0.15-0.4 Hz) (HF), and **(C)** daytime 4-h ln ratio of absolute power of the low-frequency band (0.04-0.15 Hz) (LF)-to-HF power (LF/HF). The solid black lines indicate adjusted ORs, and the dashed grey lines indicate the 95% confidence interval (CI) bands. The reference values (OR=1) were set at the 50th percentiles of lnRMSSD (3.679 ms), lnHF (5.549 ms^2^), and lnLF/HF(–0.0253), and the knots were set at the 5th, 35th, 65th, and 95th percentiles.

Dose-response relationships were evaluated using restricted cubic spline analysis based on Model 3 (Figure 1). An S-shape linear association with moderate-to-severe CSVD was suggested for daytime 4-h lnRMSSD (*P* for nonlinearity = 0.543), 4-h lnHF (*P* for nonlinearity = 0.31), and 4-h lnLF/HF (*P* for nonlinearity = 0.502). Estimated curves showed decreasing trends for daytime 4-h lnRMSSD and 4-h lnHF and increasing trends for daytime 4-h lnLF/HF.

## 4 Discussion

In this study, we observed a significant correlation between total burden of CSVD and daytime HRV but not nighttime HRV. Furthermore, there was an S-shaped dose-response linear association between total burden of CSVD and ln-transformed daytime RMSSD, HF, and LF/HF.

Previous studies of HRV in this population have mainly focused on the correlation between 24-h HRV parameters and the total burden of CSVD. In one such study, RMSSD was found to be negatively correlated with the total burden of CSVD in patients with minor stroke and transient ischemic attacks (15). Another study demonstrated that lower SDNN and RMSSD values were associated with higher total burden of CSVD in elderly patients with hypertension (29). In other research, lower SDNN was found to be associated with total burden of CSVD among diabetic patients but not among nondiabetic patients (16). In this study, no correlation was found between 24-h HRV parameters and total burden of CSVD, perhaps in part because of the small number of study participants and differences in patient characteristics.

HRV is an indicator of circadian autonomic nervous system function, which can change with aging and disease (30, 31). Although 24-h HRV has been regarded as the standard detection method, this measurement does not accurately reflect differences between daytime and nighttime HRV (32). To the best of our knowledge, the current study is the first to confirm differences in the correlation between CSVD and daytime and nighttime HRV. We found that decreased daytime lnRMSSD and lnHF and increased daytime lnLF/HF are independent predictors of total burden of CSVD. However, we did not observe any correlation between CSVD burden and daytime SDNN or LF or between CSVD burden and nighttime HRV parameters.

Two previous studies demonstrated different findings from the ones reported here. One of these studies demonstrated an association between increased nighttime RMSSD and progression of CSVD (33). However, RMSSD in this study was calculated from intermittent heart rate records of ambulatory blood pressure monitoring, and so the results need to be interpreted with caution. In the other study, lower SDNN and higher LF/HF during a nighttime sleep period were associated with moderate-to-severe WMH (Fazekas score of 2 or 3) (34). In this study, the control group consisted of individuals without CSVD MRI markers other than mild WMH (Fazekas score of 0 or 1). Because of these differences in inclusion and exclusion criteria, the results from this previous study cannot be directly compared with ours.

In previous research, an association was demonstrated between severe total burden of CSVD (3–4 point) and higher risk of cognitive impairment (35). Compared to healthy individuals, patients with cognitive impairment performing the same cognitive task will demonstrate activation across a larger range of the cerebral cortex, accompanied by a larger increase in regional cerebral blood flow (CBF) (36). The autonomic nervous system is involved in regulating CBF to rapidly maintain sufficient regional cerebral perfusion (37). The neural vascular coupling triggered by these cognitive tasks is accompanied by matching changes in HRV (38–40). Additionally, more cognitive activities will take place during daytime wakefulness than during nighttime sleep. More cognitive activities are accompanied by more frequent regulation of CBF, and are matched with more changes in autonomic nervous system activity. The different correlations between daytime and nighttime HRV and CSVD found in this study may therefore be attributed to the above factors.

Among the various time-domain and frequency-domain parameters of daytime HRV, we found that only lnRMSSD, lnHF, and lnLF/HF were associated with CSVD, with these parameters demonstrating an S-shaped dose-response linear relationship. It is generally believed that RMSSD and HF reflect parasympathetic activity, whereas LF/HF is commonly used as an index of sympatho-vagal balance (41, 42). Therefore, our findings suggested that daytime parasympathetic HRV parameters are independent influencing factors of total burden of CSVD and may serve as potential therapeutic observation indicators for CSVD. It is important to acknowledge that the aforementioned findings are derived from a cross-sectional analysis of the study population, which does not allow for a straightforward inference that parasympathetic HRV parameters are predictive of the progression of CSVD. Furthermore, this conclusion is based on the comparison of average values between two distinct groups. It is also essential to recognize that HRV parameters can fluctuate significantly within the same individual across different days of assessment. Therefore, the conclusions drawn from this study are not directly transferable for making comparisons between different individuals.

Real-time regulation of CBF is a complex process that depends on the synergistic effect of various intrinsic factors, such as the autonomic nervous system, smooth muscle cells, myogenic mechanisms, and cerebral metabolism, along with systemic factors like arterial blood pressure, blood gases, and ventilation (37, 39). Dysregulated neurovascular coupling (43, 44), impaired cerebrovascular reactivity (8), and elevated arterial stiffness (9) are associated with the severity of CSVD, which suggesting that more severe CSVD is accompanied by poorer intrinsic regulation of CBF. Consequently, in individuals with severe CSVD, systemic factors mediated by the sympathetic nervous system are likely to play a more significant role in the regulation of CBF when facing daily cognitive demands. In a typical healthy human heart, an escalation in sympathetic activity is typically accompanied by a reduction in parasympathetic activity during most daily activities (12). These elements collectively account for the observed correlation between daytime parasympathetic HRV parameters and the total burden of CSVD identified in our study.

This study had several limitations. First, the participants in this study were all recruited from a single cerebrovascular disease clinic and had suffered a nondisabling ischemic cerebrovascular event 3 months ago. Therefore, our findings may not be applicable to all patients with CSVD. Second, our findings were derived from cross-sectional investigation; we were thus unable to explore a potential causal relationship between parasympathetic dysfunction and CSVD. Third, it should be noted that nondisabling ischemic cerebrovascular event can have diverse etiologies. However, this study did not include high-resolution MRI or other specialized cerebrovascular assessments, so etiological classification could not be performed. Last, to assess HRV during sleep, we selected 11 PM to 3 AM as the nighttime analysis time period, and we selected a time period of the same duration during the day to meet the specifications for analysis. Because of this design, our findings represent only a portion of the differences between daytime and nighttime HRV parameters. In future research, we plan to incorporate motion sensors that will allow us to more accurately distinguish between activity and sleep.

## 5 Conclusion

In this study, we found that partial daytime ln-transformed HRV parameters, including increased RMSSD and HF and decreased LF/HF, demonstrated an S-shaped linear association with total burden of CSVD. However, such an association was not observed between CSVD burden and nighttime HRV parameters. These findings suggest that daytime parasympathetic HRV parameters may be independent influencing factors of total burden of CSVD and thus could serve as potential therapeutic observation indicators for CSVD.

## Data Availability

The original contributions presented in the study are included in the article/Supplementary Material, further inquiries can be directed to the corresponding author.
